# Dimensions of quality of life of older adults in relation to selected sociodemographic variables—a prospective cohort study

**DOI:** 10.3389/fpubh.2024.1419008

**Published:** 2024-09-19

**Authors:** Mariola Głowacka, Zofia Sienkiewicz, Grażyna Dykowska, Beata Haor

**Affiliations:** ^1^Collegium Medicum, The Mazovian University in Płock, Płock, Poland; ^2^Faculty of Health Sciences, Medical University of Warsaw, Warsaw, Poland; ^3^Department of Health Economics and Medical Law, Faculty of Health Sciences, Medical University of Warsaw, Warsaw, Poland; ^4^Neurological and Neurosurgical Nursing Department, Faculty of Health Science, Collegium Medicum in Bydgoszcz, Nicolaus Copernicus University in Torun, Bydgoszcz, Poland

**Keywords:** quality of life, HRQoL, older adults, pre-older adults, aging population, EQ-5D-3L

## Abstract

**Background:**

Based on its definition of an individual’s quality of life, the World Health Organization identified the following six basic domains of this concept: physical domain, psychological domain, level of independence, social relationships, environment and spirituality. The aim of the study was to examine these quality of life dimensions in pre-older and older adults in relation to selected sociodemographic variables.

**Methods:**

The study included 2,040 adults aged 55 or older. It was conducted using a diagnostic survey by means of a questionnaire with sociodemographic questions and the EQ-5D-3L descriptive system.

**Results:**

The vast majority of male and female respondents reported no problems in most EQ-5D-3L dimensions. However, the mean EQ-5D-3L index score indicated a slight difference in scores between men and women. A statistically significant difference between male and female respondents in individual dimension scores was found only for the usual activities dimension. The proportion of respondents reporting no problems decreased with age in most dimensions, except for anxiety/depression. Age was statistically significantly correlated with all individual dimension scores and the index score. The vast majority of respondents across all education levels reported no problems in most dimensions. Education was statistically significantly correlated with all individual dimension scores and the index score.

**Conclusion:**

The vast majority of respondents across both sexes and all education levels reported no problems in most EQ-5D-3L dimensions. The proportion of respondents reporting no problems decreased with age in most EQ-5D-3L dimensions. Systematic research on dimensions of health-related quality of life will help design measures for healthy and successful ageing.

## 1 Introduction

Interest in quality of life (QoL) dates back to ancient times. The term ‘quality’, understood as a certain degree of perfection, was first used by Plato. The philosopher emphasised a subjective approach to quality, indicating that it can only be understood through experience (1, 2).

Today, QoL is of interest to researchers in many areas of science and social policy. The value of life is determined, among other things, by its quality, which implies a certain standard—a reference point for human life (3, 4). In medicine, the standardisation of the term ‘quality of life’ was prompted by the definition proposed by the World Health Organization (WHO) in 1948, which states that health is “a state of complete physical, mental and social wellbeing not merely the absence of disease…” (5–8). The definition went beyond the previous traditional biomedical approach to health and leaned towards a biopsychosocial approach to measures of health (7).

One of the first definitions of QoL was developed in 1972 by Rourke and Dalkey, who identified its two key elements, namely satisfaction with life and happiness (9, 10). Subsequent definitions evolved and defined QoL in broader terms. One of the classifications organising the existing definitions of QoL in the medical context is that by Morag Farquhar. The author classified QoL definitions into ‘expert’ or ‘professionals’ definitions and ‘lay’ definitions (11). ‘Expert’ definitions were further classified into global definitions (type 1), component definitions (type 2), focused definitions (type 3) and combination definitions (type 4). Global definitions encompass all aspects of QoL and may incorporate ideas relating to overall satisfaction with life, wellbeing in different areas of life and happiness. Component definitions break down global QoL into a number of component parts (subjective and objective). Focused definitions relate only to specific components of QoL, e.g., health. Here, the most commonly addressed concept is that of the relationship between QoL and health status. Combination definitions combine elements of both global and component definitions. The second group of definitions, i.e., lay definitions, suggest that QoL is such a subjective concept that it cannot be captured in the form of standards or norms (9–12). The WHO definition of QoL, which states that quality of life is “an individual’s perception of their position in life in the context of the culture and value systems in which they live and in relation to their goals, expectations, standards and concerns” is a combination definition (5). It combines elements of both global and component definitions and incorporates additional domains and dimensions such as the individual’s external environment, expectations and economic conditions (13).

The definition of QoL has evolved following a number of studies carried out since the beginning of the 1960s and 1970s, which were pioneered by Campbell. In 1971, together with Converse and Rodgers, he carried out research among US populations aimed at assessing their perceived satisfaction with life. The researchers designed a composite measure of global sense of wellbeing that considered a number of specific domains of life. These included: marriage, family life, health, neighbours, housework, professional career, life in the US, residential environment, education and standard of living (2, 13). Based on his study, Campbell found that there is no clear relationship between the objective parameters of QoL and the level of satisfaction with life (14).

The WHO definition of health, underlining biopsychosocial wellbeing, rather than mere absence of disease, and research demonstrating that objective health status is not a straightforward indicator of needs satisfaction and happiness prompted researchers to further develop the concept of QoL in medical sciences. It was then that health-related quality of life (HRQoL) was defined. One of the first definitions of HRQoL was proposed by Shipper, who stated that it is the impact of disease and its treatment on daily functioning and overall satisfaction with life as perceived by the patient (15). According to this author, HRQoL covers four basic dimensions: physical condition and mobility, mental condition, social and financial situation and somatic sensations (12, 15).

Nowadays, we are witnessing a rapid development of tools used to assess overall QoL and its dimensions. Those most commonly used are generic, specific and mixed questionnaires. Generic questionnaires can be used to assess HRQoL in both healthy individuals and those with health conditions. Unfortunately, they are usually not very sensitive to changes resulting from the treatment used for a given condition. Specific questionnaires are designed for use in a specific group of patients and are more sensitive to changes in health status. Mixed questionnaires include elements of generic questionnaires, but are specific to a given condition (16).

In 1991, the first international quality of life group was established within the WHO. Its work focused mainly on different dimensions of HRQoL (16). In 1994, experts from 15 centres in different countries developed and published a quality of life assessment instrument - the WHOQOL-100 - based on the WHO definition of quality of life. The WHOQOL-100 shows good psychometric properties, as indicated by the results of international multi-centre studies. WHO experts also developed the WHOQOL-BREF, which is an abbreviated version of the WHOQOL-100 (5, 6, 9, 17, 18). Unfortunately, the two scales proved to be less useful in research among older individuals. As compared to younger people, seniors attach importance to other aspects of life and view social relationships and mental wellbeing in a different way (19). Therefore, it is necessary to adapt the existing HRQoL assessment instruments for use in older adults to identify their priority problems and determinants of their satisfaction with medical care (20). One such scale intended for use in older individuals is the WHOQOL-OLD Module. However, it is recommended to be used together with either the WHOQOL-100 or the WHOQOL-BREF. Another tool was also developed, namely the WHOQOL-AGE, which is an optimal instrument for assessing QoL in ageing populations (19–21). An instrument recommended by the National Institute for Health and Care Excellence (NICE) is the EQ-5D-3L questionnaire developed by the EuroQoL Group, which consists of a system of 5 dimensions and 3 levels (18, 22, 23).

The aim of this study was to analyze different dimensions of quality of life in pre-older and older adults in relation to selected sociodemographic variables. We hypothesized that: (1) age would be significantly associated with quality of life as measured by the EQ-5D-3L, with older adults reporting lower scores across multiple dimensions; (2) educational level would significantly influence quality of life, with higher education levels correlating with better outcomes; and (3) gender differences would emerge, particularly in the anxiety/depression dimension, with women reporting more problems than men.

## 2 Materials and methods

### 2.1 Participants

The study was carried out among pre-old, young-old and oldest-old adults living in Płock, Poland. The inclusion criteria were as follows: age 55 or older, permanent residence in Płock, absence of cognitive impairment as assessed by the Mini Mental State Examination (MMSE). Respondents were recruited using purposive sampling. Information about the study was promoted by the Mazovian Academy and communicated in every primary healthcare center (POZ) in Płock. During the study period, every individual visiting these centers was informed about the research and the possibility to participate, ensuring broad awareness and access to potential participants. Additionally, members of the University of the Third Age in Płock were also invited to participate. The recruitment process had three stages. In the first stage, respondents were informed about the purpose of the study and its anonymous and voluntary nature. A total of 2,253 people gave their consent to participate in the study. In the next stage, respondents completed relevant questionnaires in paper form or electronically on the LimeSurvey platform (LimeSurvey GmbH, Hamburg, Germany). In the third stage, the completed questionnaires were verified. Ultimately, a total of 2,040 fully completed surveys were analysed. Participants were then categorized into the following age groups: pre-old adults (55–60 years), young-old adults (61–75 years), middle-old adults (76–90 years), and oldest-old adults (over 90 years). The term ‘pre-old’ was used to describe the pre-retirement age group, defined in accordance with Polish law (24). Language correction of the text was performed using the generative AI tool ChatGPT, based on the GPT-4 model, provided by OpenAI.

### 2.2 Measures

The study was conducted using a diagnostic survey by means of the EQ-5D-3L questionnaire, which was used with the permission of the EuroQoL Research Foundation. Respondents were also asked to complete a sociodemographic questionnaire developed by the authors of the present study.

The EQ-5D questionnaire was designed by the EuroQoL Group to assess HRQoL. It consists of two parts: the EQ-5D descriptive system and the EQ visual analogue scale (EQ VAS). The questionnaire can be completed as follows: in paper form by respondents themselves; in paper form by the interviewer during a face-to-face interview with the respondent; digitally by respondents (an Internet application), or; through a phone call with the interviewer (25–28).

There are three versions of the EQ-5D: EQ-5D-5L, EQ-5D-3L and EQ-5D-Y. The EQ-5D-5L comprises 5 dimensions, each of which has 5 levels, whereas the EQ-5D-3L and the EQ-5D-Y comprise 5 dimensions, each of which has 3 levels (18, 22, 23).

The EQ-5D-3L is recommended for use in adults. It can be used in studies in individual patients or populations in order to compare the health states of patients in a given clinical situation with QoL of the general population (26, 29–33).

The EQ-5D-3L descriptive system comprises the following 5 dimensions: mobility, self-care, usual activities, pain/discomfort and anxiety/depression. Each dimension has three levels coded as one-digit numbers: no problems—coded as 1, some problems—coded as 2, and extreme problems—coded as 3. Respondents are asked to select one level for each dimension. The digits for five dimensions can be combined into a 5-digit number describing the respondent’s health state. The 5-digit number can be converted into a single summary index by applying a formula that attaches weights to each level in each dimension. The weights represent the preferences of the general population of a country/region (26, 29). The EQ VAS is a visual analogue scale that records the patient’s self-rated health on the day it is administered (26, 28).

The study presents data derived from the analysis of the sociodemographic questionnaire and the EQ-5D-3L descriptive system results.

### 2.3 Procedure and ethical considerations

The study was conducted following the recommendations and was reviewed and approved by the Bioethics Committee of the Mazovian Academy in Plock (statute no. KB/N/BN/P/1.2021). All participants gave their written informed consent in accordance with the Declaration of Helsinki.

### 2.4 Statistical analysis

The results were analysed descriptively, graphically and statistically. Statistica 10.0 (StatSoft Polska Sp. z o.o., Kraków, Poland) and PQStat (PQStat Software, Poznań, Poland) were used to analyse the data. Statistical significance was set at *p* < 0.05. Relationships between quantitative variables were assessed using Pearson’s correlation coefficients. The ANOVA test and the Student’s t-test were used to examine the statistical relationships between the features analysed. Descriptive statistics for continuous variables were reported as means (M), standard deviations (SD), results of the analysis of variance (F), results of the Student’s *t*-test (*t*), medians (Me), minimum values (Min.), maximum values (Max.), Q_25_ (lower quartile) and Q_75_ (upper quartile), confidence intervals, correlation coefficients (*r*) and degrees of freedom (df). Categorical variables were reported as frequencies (*N*).

## 3 Results

The study included 2,040 respondents, whose mean age was 65.4 years. The majority of respondents were women (68.9%; *n* = 1,406). Men accounted for 31.1% (*n* = 634) of respondents.

Table 1 shows EQ-5D-3L dimension results by sex.

**Table 1:** EQ-5D-3L dimensions by sex.

Sex		Female		Male		Total	
Dimension	Response	*n*	%	*n*	%	*n*	%
Mobility	No problems (1)	1,097	78.0	496	78.2	1,593	78.1
	Some problems (2)	300	21.3	132	20.8	432	21.2
	Confined to bed (3)	9	0.6	6	0.9	15	0.7
Self-care	No problems (1)	1,267	90.1	552	87.1	1819	89.2
	Some problems (2)	128	9.1	77	12.1	205	10.0
	Unable (3)	11	0.8	5	0.8	16	0.8
Usual activities	No problems (1)	1,060	75.4	432	68.1	1,492	73.1
	Some problems (2)	336	23.9	195	30.8	531	26.0
	Unable (3)	10	0.7	7	1.1	17	0.8
Pain/discomfort	No (1)	584	41.5	279	44.0	863	42.3
	Moderate (2)	812	57.8	351	55.4	1,163	57.0
	Extreme (3)	10	0.7	4	0.6	14	0.7
Anxiety/depression	No (1)	905	64.4	422	66.6	1,327	65.0
	Moderate (2)	487	34.6	209	33.0	696	34.1
	Extreme (3)	14	1.0	3	0.5	17	0.8

(1)—level 1, (2)—level 2, (3)—level 3; *n*—number of respondents.

The vast majority of respondents reported no problems (level 1) with self-care (*n* = 1,819; 89.2%), mobility (*n* = 1,593; 78.1%) and the ability to perform usual activities (*n* = 1,492; 73.1%) and reported no anxiety or depression (*n* = 1,327; 65%). However, most respondents (*n* = 1,163; 57%) reported having moderate pain or discomfort (level 2). The proportion of respondents selecting level 3 was highest for self-care (*n* = 16; 0.8%), usual activities (*n* = 17; 0.8%) and anxiety/depression (*n* = 17; 0.8%) and lowest for mobility (*n* = 15; 0.7%) and pain/discomfort (*n* = 14; 0.7%).

The proportion of female respondents selecting no problems (level 1) was highest for self-care (*n* = 1,267; 90.1%) and usual activities (*n* = 1,060; 75.4%). The proportion of female respondents selecting level 3 was lowest for the mobility dimension - only 9 female respondents (0.6%) were confined to bed - and highest for the anxiety/depression dimension (*n* = 14; 1.0%). Men were more likely than women to report no problems with mobility (*n* = 496; 78.2%), anxiety/depression (*n* = 422; 66.6%) and pain/discomfort (*n* = 279; 44.0%). The proportion of male respondents selecting level 3 was lowest for anxiety/depression (*n* = 3; 0.5%) and highest for usual activities (*n* = 7; 1.1%).

Table 2 shows mean index scores for male and female respondents.

**Table 2:** Index scores by sex.

Sex	*n*	Mean	SD	Confidence −95.0%	Confidence +95.0%	Min.	Max.	Q25	Median	Q75
Female	1,406	0.888	0.127	0.881	0.895	−0.523	1.000	0.822	0.894	1.000
Male	634	0.886	0.132	0.876	0.896	−0.523	1.000	0.822	0.894	1.000
Total	2040	0.887	0.129	0.882	0.893	−0.523	1.000	0.822	0.894	1.000

The mean index score obtained by respondents was 0.887. The standard deviation was more than 14% of the mean, indicating a slight difference in scores between male and female respondents. Women had a slightly higher mean index score (0.888) compared to men (0.886). Differences in EQ-5D-3L scores between male and female respondents are shown in Table 3.

**Table 3:** Differences in EQ-5D-3L scores between male and female respondents.

Dimension	Mean score: female	Mean score: male	*t*	df	*p*	Valid *N*: female	Valid *N*: male	SD: female	SD: male	Quotient *F* Variance	*p* Variance
Mobility	1.226	1.227	−0.046	2038	0.963	1,406	634	0.434	0.441	1.036	0.592
Self-care	1.107	1.137	−1.856	2038	0.064	1,406	634	0.333	0.367	1.210	0.004
Usual activities	1.253	1.330	−3.440	2038	0.001	1,406	634	0.451	0.493	1.197	0.007
Pain/discomfort	1.592	1.566	1.052	2038	0.293	1,406	634	0.506	0.509	1.010	0.872
Anxiety/depression	1.366	1.339	1.144	2038	0.253	1,406	634	0.502	0.484	1.078	0.273
Index score	0.888	0.886	0.309	2038	0.757	1,406	634	0.127	0.132	1.083	0.232

A statistically significant difference (*p* < 0.05) in scores between male and female respondents was found only for the usual activities dimension. The mean age of female respondents was 64.8 years, whereas the mean age of male respondents was 66.87 years. Over half of respondents were aged between 61 and 75 years (*n* = 1,073; 52.6%). The smallest proportion of respondents were aged over 90 (*n* = 24; 1.2%). The study also included respondents aged 60 or under (*n* = 664; 32.5%) and those aged between 76 and 90 (*n* = 279; 13.7%). Table 4 shows EQ-5D-3L dimension results by age group.

**Table 4:** EQ-5D-3L dimension results by age group.

Age		60 or under		61–75		76–90		over 90	
Dimension	Level	*n*	%	*n*	%	*n*	%	*n*	%
Mobility	No problems (1)	587	88.4	858	80.0	143	51.3	5	20.8
	Some problems (2)	75	11.3	206	19.2	134	48.0	17	70.8
	Confined to bed (3)	2	0.3	9	0.8	2	0.7	2	8.3
Self-care	No problems (1)	646	97.3	971	90.5	191	68.5	11	45.8
	Some problems (2)	16	2.4	97	9.0	81	29.0	11	45.8
	Unable (3)	2	0.3	5	0.5	7	2.5	2	8.3
Usual activities	No problems (1)	602	90.7	749	69.8	132	47.3	9	37.5
	Some problems (2)	57	8.6	318	29.6	143	51.3	13	54.2
	Unable (3)	5	0.8	6	0.6	4	1.4	2	8.3
Pain/discomfort	No (1)	385	58.0	406	37.8	67	24.0	5	20.8
	Moderate (2)	277	41.7	659	61.4	210	75.3	17	70.8
	Extreme (3)	2	0.3	8	0.7	2	0.7	2	8.3
Anxiety/depression	No (1)	514	77.4	674	62.8	127	45.5	12	50.0
	Moderate (2)	146	22.0	390	36.3	149	53.4	11	45.8
	Extreme (3)	4	0.6	9	0.8	3	1.1	1	4.2

(1)—level 1, (2)—level 2, (3)—level 3; *n*—number of respondents.

In the mobility, self-care, usual activities and pain/discomfort dimensions, the proportion of respondents selecting ‘no problems’ (level 1) decreased with age. A different trend was seen for the anxiety/depression dimension among the oldest age groups. Respondents aged over 90 were more likely (*n* = 12; 50%) than respondents aged between 76 and 90 (*n* = 127; 45.5%) to report no problems in this dimension. In the mobility, self-care and usual activities dimensions, the proportion of respondents reporting some problems (level 2) increased with age. A different trend was seen for the pain/discomfort and anxiety/depression dimensions among the oldest age groups—respondents aged over 90 were less likely to report having moderate pain/discomfort (70.8%) and being moderately anxious/depressed (45.8%) than respondents aged between 76 and 90 (75.3 and 53.4% respectively). In all dimensions, respondents aged over 90 were the most likely to select level 3, indicating extreme problems/an inability to perform. Table 5 shows mean index scores by age group.

**Table 5:** Index scores by age group.

Age	*n*	Mean	SD	Confidence −95.0%	Confidence +95.0%	Min.	Max.	Q25	Median	Q75
Under 60	664	0.929	0.103	0.921	0.937	−0.523	1.000	0.894	1.000	1.000
60–75	1,073	0.883	0.120	0.875	0.890	−0.523	1.000	0.822	0.894	1.000
75–90	279	0.821	0.140	0.805	0.838	−0.523	1.000	0.768	0.822	0.894
Over 90	24	0.706	0.332	0.566	0.846	−0.523	1.000	0.716	0.766	0.842

The highest mean index scores were reported by respondents aged under 60 (0.929) and those aged between 60 and 75 (0.883), whereas the lowest mean index score was reported by respondents aged over 90 (0.706). Table 6 shows the correlation between age and EQ-5D-3L dimension results and the index score.

**Table 6:** Correlation between age and EQ-5D-3L results.

Item	Mobility	Self-care	Usual activities	Pain/discomfort	Anxiety/depression	Index score
*r*	0.287	0.296	0.320	0.239	0.210	0.302
*p*	0.000	0.000	0.000	0.000	0.000	0.000

A statistically significant, moderate correlation was found between age and the usual activities dimension score and The index score. There was also a statistically significant, small correlation between age and the mobility, self-care, pain/discomfort and anxiety/depression dimensions. Of the respondents, 8.6% (*n* = 175) had primary education, 27.8% (*n* = 567) had basic vocational education and 23.8% (*n* = 486) had tertiary education. The largest proportion of respondents (*n* = 812; 39.8%) had secondary/post-secondary education. Table 7 shows EQ-5D-3L dimension results by education level.

**Table 7:** EQ-5D-3L dimensions by education level.

Education		Primary or lower		Vocational		Secondary/post-secondary		Tertiary	
Dimension	Level	*n*	%	*n*	%	*n*	%	*n*	%
Mobility	No problems (1)	105	60.0	446	78.7	623	76.7	419	86.2
	Some problems (2)	64	36.6	117	20.6	186	22.9	65	13.4
	Confined to bed (3)	6	3.4	4	0.7	3	0.4	2	0.4
Self-care	No problems (1)	128	73.1	496	87.5	741	91.3	454	93.4
	Some problems (2)	41	23.4	68	12.0	68	8.4	28	5.8
	Unable (3)	6	3.4	3	0.5	3	0.4	4	0.8
Usual activities	No problems (1)	100	57.1	394	69.5	593	73.0	405	83.3
	Some problems (2)	71	40.6	169	29.8	213	26.2	78	16.0
	Unable (3)	4	2.3	4	0.7	6	0.7	3	0.6
Pain/discomfort	No (1)	57	32.6	215	37.9	319	39.3	272	56.0
	Moderate (2)	113	64.6	346	61.0	493	60.7	211	43.4
	Extreme (3)	5	2.9	6	1.1	0	0.0	3	0.6
Anxiety/depression	No (1)	103	58.9	358	63.1	513	63.2	353	72.6
	Moderate (2)	67	38.3	201	35.4	298	36.7	130	26.7
	Extreme (3)	5	2.9	8	1.4	1	0.1	3	0.6

(1)—level 1, (2)—level 2, (3)—level 3; *n*—number of respondents.

The vast majority of respondents across all education levels reported no problems (level 1) in the mobility, self-care, usual activities and anxiety/depression dimensions. However, most respondents across most education levels reported having moderate pain/discomfort (level 2). The exception were respondents with tertiary education. More than half of them (*n* = 272; 56%) reported no pain or discomfort (level 1).

In all dimensions, respondents with primary or lower education were the most likely to select level 2, indicating some problems, or level 3, indicating extreme problems/inability to perform. Table 8 shows mean index scores by education level.

**Table 8:** Index scores by education level.

Education	*n*	Mean	SD	Confidence −95.0%	Confidence +95.0%	Min.	Max.	Q25	Median	Q75
Primary or lower	175	0.821	0.204	0.790	0.851	−0.523	1.000	0.770	0.848	0.899
Basic vocational	567	0.879	0.138	0.867	0.890	−0.523	1.000	0.822	0.894	1.000
Secondary/post-secondary	812	0.890	0.098	0.883	0.897	0.147	1.000	0.822	0.894	1.000
Tertiary	486	0.917	0.118	0.906	0.927	−0.342	1.000	0.868	0.925	1.000

The highest mean index scores were reported by respondents with tertiary education (0.917) and those with secondary/post-secondary education (0.8903) and the lowest mean index score was reported by respondents with primary or lower education (0.821). Table 9 shows the correlation between education and EQ-5D-3L dimension scores and the index score.

**Table 9:** Correlation between education and EQ-5D-3L results.

Item	Mobility	Self-care	Usual activities	Pain/discomfort	Anxiety/depression	Index score
*r*	−0.138	−0.148	−0.154	−0.145	−0.088	−0.179
*p*	0.000	0.000	0.000	0.000	0.000	0.000

A statistically significant, small correlation was found between education and scores for the mobility, self-care, usual activities and pain/discomfort dimensions and the index score. A statistically significant, very small correlation was found for scores for the anxiety/depression dimension.

## 4 Discussion

### 4.1 Dimensions of quality of life in pre-older and older adults

Demographic ageing, which is also common to populations across Poland, carries a number of implications which have an impact on QoL. The systematic rise in the proportion of older people in the population, falls in fertility rates, growth in life expectancy and the “double ageing” of the population pose major challenges for social policies and health systems in terms of satisfying the needs of senior citizens (20, 34). However, today’s older adult individuals in Poland live the life of old age in a new way. Their lifestyle is changing with regard to three aspects, namely education, consumption and activity. They are increasingly educated, have digital skills and communicate electronically. They often use cultural, recreational, tourist and educational services. Moreover, they have social networks other than their family and neighbours and take part in social, active citizenship and voluntary activities (20). Thus, they engage in healthy, successful and active ageing. Healthy ageing, successful ageing and active ageing are new concepts which have changed perceptions of QoL in older people (20, 35).

Based on its definition of QoL, the WHO identified the following six basic domains of this concept: physical domain, psychological domain, level of independence, social relationships, environment and spirituality (beliefs, religion) (5, 36). According to the Polish Agency for Health Technology Assessment and Tariff System, the EQ-5D (EQ-5D-3L or EQ-5D-5L) questionnaire is the preferred instrument for measuring QoL in adults due to its common use, which favours the comparability of results (27). The vast majority of male and female respondents in the present study reported no problems in most EQ-5D-3L dimensions. However, most respondents reported having moderate pain or discomfort. The proportion of respondents reporting extreme problems was highest for the self-care, usual activities and anxiety/depression dimensions and lowest for mobility and pain/discomfort. The proportion of female respondents reporting no problems was highest for the self-care and usual activities dimensions, whereas the proportion of female respondents reporting extreme problems was highest for the anxiety/depression dimension and lowest for mobility. Most male respondents reported no problems with mobility, anxiety/depression and pain/discomfort. The proportion of male respondents reporting extreme problems was highest for usual activities and lowest for anxiety/depression. However, the mean index score indicated a slight difference in scores between male and female respondents. A statistically significant difference between male and female respondents in individual dimension scores was found only for the usual activities dimension. The proportion of respondents reporting no problems decreased with age in most dimensions, except for anxiety/depression. In the mobility, self-care and usual activities dimensions, the proportion of respondents reporting some problems increased with age. Respondents aged over 90 were the most likely to report extreme problems/inability to perform. This was also confirmed by the fact that they had the lowest mean index score. Age was statistically significantly correlated with all individual dimension scores and the index score. The vast majority of respondents across all education levels reported no problems in most EQ-5D-3L dimensions. This did not apply to pain/discomfort, with most participants reporting moderate problems in this dimension, except for respondents with tertiary education, most of whom reported no pain or discomfort. In all dimensions, respondents with primary or lower education were the most likely to report some or extreme problems. They also had the lowest mean index score. Education was statistically significantly correlated with all individual dimension scores and the index score. A pilot study by Golicki et al. carried out in 2008 among adult Poles to elicit the EQ-5D value set for Poland showed that the predominating problems were pain/discomfort (40.1%) and anxiety/depression (37.8%). The study group was least likely to report problems with self-care (3.3%). A total of 16.8% of respondents had problems with mobility and 13.8% with performing usual activities. The respondents in the study tended to be female, employed and in relationships. They also tended to be living in urban areas and have secondary or tertiary education. Their mean age was 42.8 years (37). In a study by Golicki and Niewada carried out in 2014 among adult Poles to derive population norms for the EQ-5D-3L in Poland, the vast majority of respondents reported no problems in any of the dimensions. The respondents tended to be female, employed and have secondary education. They more often lived in urban rather than rural areas and their mean age was 48.3 years. The proportion of participants reporting some problems was highest for pain/discomfort (43.2%) and anxiety/depression (31.9%). Extreme problems were most frequently reported for pain/discomfort (2.6%), anxiety/depression (1.5%) and usual activities (1.7%). EQ-5D-3L index scores were found to significantly decrease with age and were lower in women (38). In a study by Golicki et al. aimed at deriving a Polish utility tariff for EQ-5D-5L health states, the vast majority of respondents reported problems relating to pain/discomfort (53.4%) and anxiety/depression (42.9%). A total of 28.5% of respondents reported problems with mobility and 20.6% had problems with performing usual activities. The smallest proportion of respondents reported problems with self-care (9.9%). The study was carried out among adults, who tended to be female, employed, living in urban areas and have secondary education (39). Another study by Golicki et al. was aimed at estimating a regional EQ-5D-3L population norm for Central and Eastern Europe (citizens of Poland, Hungary and Slovenia). The largest proportions of respondents from these countries reported problems relating to pain/discomfort (42.7%) and anxiety/depression (33.0%). The smallest proportion of respondents reported problems with self-care (7.4%). Problems with mobility and usual activities were reported by 17.3 and 22.2% of respondents, respectively. In all five EQ-5D-3L dimensions, problems were most frequently reported by Slovenian respondents, followed by Poles and Hungarians. Education was found to significantly influence the occurrence of health problems in all EQ-5D-3L dimensions, which were most frequently reported by respondents with the lowest level of education. Index scores differed by sex and age (moderately higher in men) (40). In the *PolSenior2* study carried out among older people in Poland using the WHOQOL-AGE scale, among other instruments, 59.9% of respondents rated their QoL as good and only 3.3% rated it as poor or very poor. Most respondents were satisfied with their health (62.4%) and their ability to perform the activities of daily living (77.0%). Male respondents had a slightly higher mean QoL score compared with female respondents. The mean QoL score was highest for the youngest group of respondents (aged 60–65) and lowest for respondents in the oldest age group (85 or over). Respondents living in rural areas reported lower QoL compared to those living in urban areas. QoL was highest in participants with higher education and lowest in those with primary education (20). The results of the VES-13 scale used in the *PolSenior2* study showed that almost 40% of respondents aged 60 or over scored 3 points or higher on the scale and were thus deemed to need comprehensive geriatric assessment. Those respondents were significantly more likely to be affected by major geriatric syndromes. Women, respondents with primary or lower education and those living in rural areas had significantly higher VES-13 scores (41). The *PolSenior2* study also included an assessment of the functional status of respondents. It was found that the proportion of participants reporting IADL (Instrumental Activities of Daily Living) deficits increased with age, especially among women. Respondents with primary education and those living in rural areas were more likely than other groups to report IADL deficits. It was also found that most respondents had no impairments in activities of daily living (ADL) and that the proportion of respondents with ADL impairments increased with age, especially among women (42). The *PolSenior2* study also found that 47.6% of respondents aged 60 or over experienced chronic pain. It was more common in women, respondents with primary education, manual workers and respondents living in rural areas (20). Moreover, the *PolSenior2* study found that almost one in four older people in Poland show depressive symptoms and that their prevalence increased with age. According to the study, depressive symptoms are more common in women, people with a low level of education, people in a poor financial situation, residents of rural areas, people who need help from others and people who feel lonely (20). The quality of life of older people is determined by a number of complex factors. A study carried out among older people in older adult homes in Malaysia reported the following QoL determinants: sex, age, education level, financial situation, physical activity and participation in outdoor leisure activities, type of accommodation, comorbidities and social support (35, 42).

### 4.2 Limitations

The present study has certain limitations. It recruited men and women living in only one town. Therefore, it is difficult to apply its results to the general population of older people, including those living in rural areas. In addition, the majority of the respondents surveyed were women. However, it is important to take into account the feminisation of old age and demographic ageing, which are not only specific to Poland. Nevertheless, these limitations offer a good starting point for further research investigating QoL dimensions in pre-older and older people in relation to sociodemographic variables.

## 5 Conclusion

The vast majority of respondents across both sexes and all education levels reported no problems in most EQ-5D-3L dimensions, with the exception of the pain/discomfort dimension, where moderate problems were commonly reported. The proportion of respondents reporting no problems decreased with age, particularly in dimensions related to mobility, self-care, and usual activities, highlighting the increased challenges faced by older adults. Additionally, significant gender differences were observed, particularly in the anxiety/depression and usual activities dimensions, suggesting a need for targeted health interventions, especially focused on mental health support for women. Education was another important factor, with higher education levels being associated with better quality of life outcomes across all dimensions. These findings underline the importance of considering sociodemographic variables, such as age, gender, and education, when designing interventions to improve quality of life in older adults. Future research should focus on expanding the scope of the study to include populations from rural areas and other regions of Poland, as well as further investigating the impact of educational interventions on improving quality of life among older adults.

## Data Availability

The raw data supporting the conclusions of this article will be made available by the authors, without undue reservation.
